# Supplementary material for: Learning curve and surgical outcome of robotic assisted colorectal surgery with ERAS program
Source: Sci Rep. 2022 Nov 29;12:20566. doi: 10.1038/s41598-022-24665-w (PMC9709162; doi:10.1038/s41598-022-24665-w)
Supplement: Supplementary file 2 — Supplementary Information 2. [file 41598_2022_24665_MOESM2_ESM.zip › RAL Raw data-Ñ[▒K/056/Patho_ñw╝╨░O▒Kñσ.pdf]

臺中榮民總醫院 臺中市臺灣大道四段1650號 病理部活體組織切片 臨床醫學實驗及病理診斷中心 檢驗(查)報告

[redacted]

Pathologic diagnosis:
1. Rectum, robotic anterior resection --- Status post endoscopic submucosal dissection with mucosal ulceration, mucin extravasation and foreign body type giant cell reaction. No residual tumor seen. The bilateral cut ends and perirectal lymph nodes (0/29) are free of tumor.
2. IMA root, dissection --- Unremarkable fibrovascular tissue and lymph nodes (0/2).
3. Rectum, cut end, distal, resection --- Chronic inflammation.

Gross description:
The specimen consists of 1) a segment of colon, 10 cm in length and 3.5 cm in proximal cut end circumference, 4 cm in distal circumference, and perirectal soft tissue. A gray tan ulcerative lesion, 1.5x0.8 cm, is noted, 1 cm to the nearest cut end and surrounded by clips. On cross section, no definite invasive mass lesion is identified grossly. The both cut ends are free. 2) IMA root, a piece of tan yellow soft tissue, 1.2x0.6x0.3 cm. 3) distal cut end, a piece of tan mucosa-covered soft tissue, 2.3x2.2x0.8 cm. Representative part for section: A) proximal cut end B) distal cut end C) colon D-F) all of ulcer G-H) specimen 2-3 J-T) lymph nodes.
#T-59600_2 #M-09413_2 0 1105 SP0000
#T-C4560_2 #M-09183_2 0 1105 000000

已電子簽章

敬告：本單僅供醫師參考使用，不具任何申請效力！！
請病患妥為保管，若不慎遺失需自負個人資料外洩責任！
